# The Treatment of Gastric Vomiting by Oxalate of Cerium

**Published:** 1906-03-03

**Authors:** 


### THE TREATMENT OF GASTRIC VOMITING BY OXALATE OF CERIUM.

Dr. Thomas Sweetnam, in the current issue of the *Dublin Journal of Medical Science*, speaks very highly of the value of the oxalate of cerium in the treatment of vomiting due to gastric disease. He observes that some years ago this salt was vaunted as a remedy for excessive vomiting during pregnancy, but fell into disrepute as inefficient for the purpose. This failure is attributed by the author to the fact that the vomiting of pregnancy is due to the movements of an enlarged uterus against the intestine, and not to any pathological alteration in the stomach itself. In the treatment of vomiting directly due to some morbid condition in the stomach the author has found cerium oxalate of the greatest value. His custom is to administer the drug in six-grain doses thrice daily in combination with ten-grain doses of the carbonate of bismuth. This combination is stated to have been successful in several instances in which bismuth salts alone had proved ineffectual. In the case of gastric ulcer the pain and vomiting are relieved almost immediately, and the same is said of the chronic catarrhal gastritis so commonly met with among ill-nourished young females. The immediate relief in these cases is followed by a substantial permanent improvement which permits of greater generosity in diet and a comparatively rapid return to a state of health. In the case of gastric carcinoma, the symptoms are greatly mitigated: hæmatemesis ceases, pain is lessened, and "patients who have been unable to retain the smallest quantity of food taken by the mouth are soon able to retain several pints of milk per diem, and also small quantities of the prepared farinaceous foods." This, of course, does not apply to instances where from the pyloric situation of the lesion the emesis depends primarily upon a mechanical obstruction of the outlet.

A case in point is described in detail, the patient being a man of forty-four years affected with pyloric carcinoma. The condition of this patient was immensely improved by a course of the mixture mentioned above, although the bismuth salt, in combination with bicarbonate of sodium, had failed to relieve him. The action of the drug, in Dr. Sweetnam's opinion, is probably the same as that of the bismuth salts, namely, that of a sedative and mild astringent to the inflamed mucous surface; but he considers it possible, if not probable, that an additional activity may reside in a trace of radio-activity. The presence of such radio-activity is hypothetical, and inferred from the fact that the cerium salt usually contains lanthanum and didymium oxalates, while, again, most of the rare earths are slightly radio-active.

